# Additive Effects of Multiple Photoprotective Mechanisms Drive Efficient Photosynthesis Under Variable Light Conditions

**DOI:** 10.1111/pce.70016

**Published:** 2025-06-17

**Authors:** Claudia Beraldo, Chiara Toffanin, Tomas Morosinotto, Alessandro Alboresi

**Affiliations:** ^1^ Department of Biology University of Padova Padova Italy; ^2^ National Biodiversity Future Center University of Palermo Palermo Italy

**Keywords:** acclimation, alternative electron transport, cyclic electron transport, flavodiiron proteins, fluctuating light, non‐photochemical quenching, photoprotection, photosynthesis

## Abstract

To cope with changing external conditions, plants undergo dynamic acclimation processes that adjust their photosynthetic machinery, optimising energy use while minimising damage to photosystems (PS). Key photoprotective mechanisms include non‐photochemical quenching (NPQ), which dissipates excess excitation energy, and alternative electron transport (AET) pathways, which prevent over‐reduction of the photosynthetic electron transport chain. This study provides a comprehensive analysis of how various photoprotective mechanisms contribute to long‐term acclimation to high and fluctuating light in *Physcomitrium patens*, a moss that exhibits well‐conserved photoprotective responses that can provide valuable insights into the adaptation of these mechanisms during evolution. Our results demonstrate that modulation of photoprotection at the level of both PSII and PSI is critical for maintaining photosynthetic efficiency and enabling acclimation to variable light conditions. *P. patens* mutants deficient in NPQ or AET, when exposed to high or fluctuating light all displayed growth defects, reduced photosynthetic efficiency and unbalanced PSI and PSII activity compared to WT plants. These findings indicate that photosynthetic response to varying light conditions depends on the complementary action of multiple protective strategies, rather than a single dominant photoprotective mechanism.

## Summary statement

This study in *Physcomitrium patens* shows that multiple photoprotective mechanisms regulating photosystems I and II have a synergic role in maintaining photosynthetic efficiency and growth under high, fluctuating light conditions.

## 1 Introduction

In photosynthetic eukaryotes, photosystems (PS) capture photons that drive linear electron flow (LEF) to produce NADPH and ATP. These energy carriers are essential for carbon fixation in the Calvin‐Benson cycle. However, under natural conditions, photosynthetic organisms are continuously exposed to fluctuating light intensities due to changes in weather, cloud cover, and canopy movements. Under these conditions, LHCs can absorb more excitation energy than the photosynthetic apparatus can use for photosynthesis. Excess energy can be harmful, leading to the formation of reactive oxygen species (ROS) and photo‐oxidative stress (Eberhard et al. 2008). Regulating light reactions helps optimise photosynthetic efficiency, allowing plants to meet their metabolic needs and maintain fitness under a highly dynamic environment (Külheim et al. 2002).

Photosynthetic organisms have evolved multiple strategies to regulate photosynthetic activity and limit photodamage. Under conditions of excess illumination, a prominent photoprotective role is played by non‐photochemical quenching (NPQ), which drives dissipation of excess excitation energy as heat (Li et al. 2009b). NPQ is activated by the decrease in lumenal pH occurring under strong light. In green algae and vascular plants, NPQ is activated by two distinct proteins, respectively, light‐harvesting complex stress‐related protein (LHCSR) and photosystem II subunit S (PSBS) (Peers et al. 2009; Niyogi and Truong 2013). While PSBS plays a prominent role in vascular plants, LHCSR is the primary NPQ activator in green algae (Bonente et al. 2011; Tibiletti et al. 2016; Redekop et al. 2020). Other eukaryotic algae like brown algae and diatoms depend on LHCX for NPQ activation, a group of proteins strictly related to LHCSR (Dittami et al. 2010). The decrease in lumenal pH also activates the xanthophyll cycle by which the violaxanthin de‐epoxidase enzyme (VDE) catalyses the de‐epoxidation of violaxanthin to zeaxanthin (Arnoux et al. 2009; Simionato et al. 2015), a carotenoid with a crucial role in NPQ (Ruban et al. 2007).

A significant contribution to photoprotection is also provided by alternative electron transport (AET) mechanisms, which prevent overreduction and damage to PSI (Allahverdiyeva et al. 2015; Shikanai and Yamamoto 2017; Burlacot 2023; Hoh et al. 2024). Cyclic electron flow (CEF) recycles electrons from PSI to the plastoquinone pool (PQ) or to Cyt *b*_*6*_ *f* complex, while pseudocyclic electron flow (PCEF) is involved in oxygen photoreduction to water downstream of PSI. Two distinct CEF pathways have been described, one depending on PGRL1/PGR5, and the other one depending on NADH dehydrogenase‐like complex (NDH). PCEF includes pathways such as the Mehler reaction and flavodiiron proteins (FLVs), the latter present in cyanobacteria, green algae, non‐vascular plants, and gymnosperms, but absent in angiosperms (Ilík et al. 2017).

All the above‐mentioned mechanisms operate short‐term, activated within minutes after a change in light intensity and have been shown to be essential for the response to fast changes in illumination (Külheim et al. 2002; Yamori and Shikanai 2016; Nawrocki et al. 2019; Burlacot 2023). However, photosynthetic organisms are often exposed to prolonged stress conditions and, in response to different environmental conditions, they also adjust their photosynthetic apparatus to optimise its efficiency and mitigate eventual photo‐oxidative stress through a process called photosynthetic acclimation (Walters 2005). Despite significant progress, the interplay and the regulation of various photoprotective mechanisms during long‐term acclimation remain under‐investigated (Eckardt et al. 2024).

In this study, we provide an integrated analysis of the strategies used by the moss *Physcomitrium patens* during long‐term acclimation to high and fluctuating light conditions. As a representative of bryophytes, which diverged from vascular plants early after land colonisation, *P. patens* can provide critical insights into the first adaptation upon transition from aquatic to terrestrial life. *P. patens* is an open field moss distributed in Europe, North America, and East Asia (Rensing et al. 2020) and in its natural habitats, it frequently encounters high light intensities and fluctuations in light availability. Understanding its strategies to cope with light stress is of great interest from both biochemical and evolutionary perspectives. In *P. patens*, NPQ is activated by both PSBS and LHCSR proteins (Alboresi et al. 2010; Beraldo et al. 2023) and, differently from angiosperms, it also expresses a full set of CEF (i.e., PGRL1/PGR5, NDH complex) (Kukuczka et al. 2014; Storti et al. 2020a) and PCEF proteins (i.e., Mehler reaction, FLVs) (Allahverdiyeva et al. 2013; Gerotto et al. 2016). The availability of a wide range of *P. patens* mutants deficient in key regulatory proteins of photosynthesis, which have been largely overlooked in acclimation studies, provides a unique opportunity to assess the relative contribution of these mechanisms under varying light conditions.

## 2 Methods

### 2.1 Plant Growth

**Figure 1:**
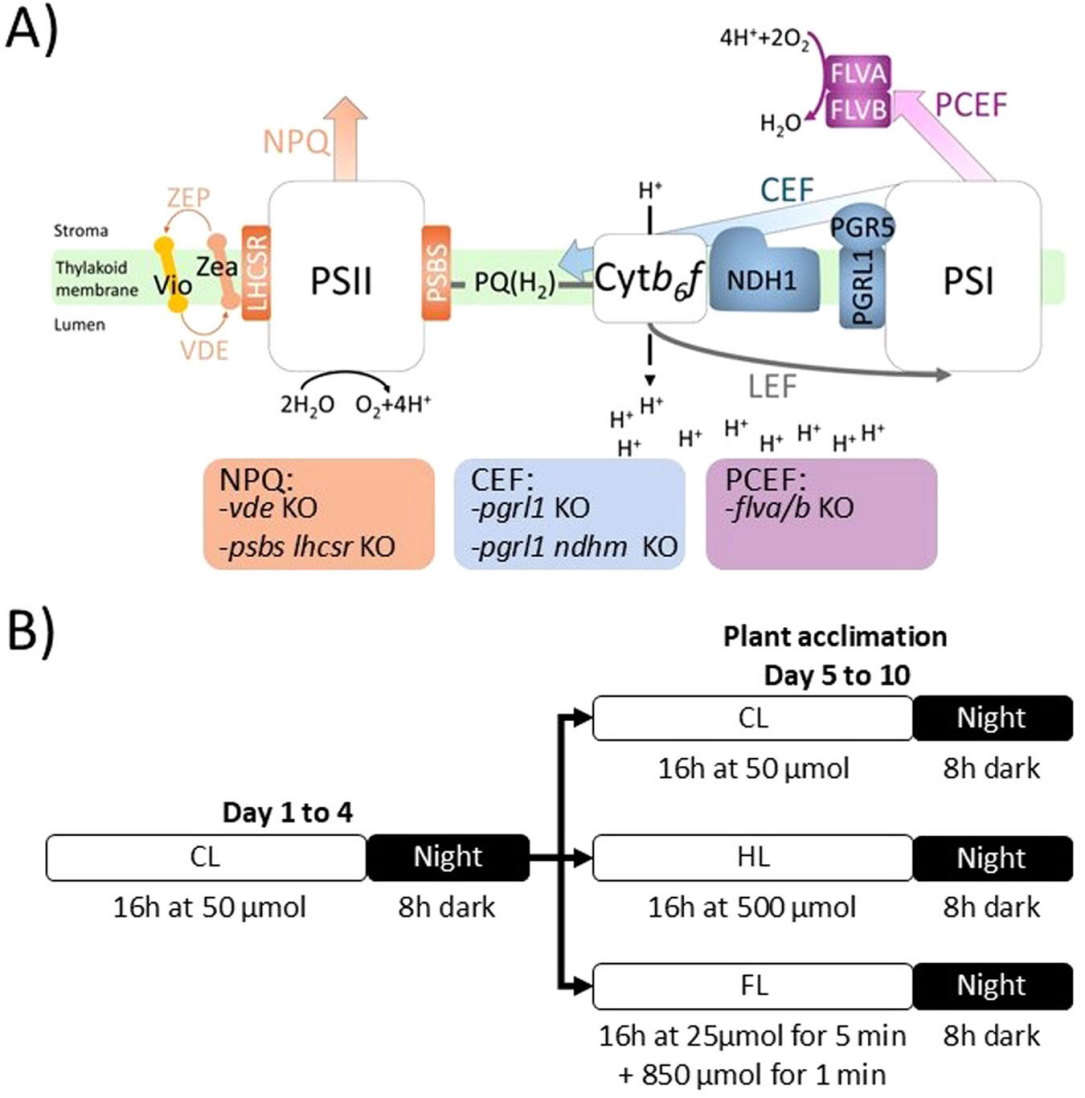
Schematic representation of *P. patens* photoprotective mutants and growth conditions used in this study. (A) Illustration of *P. patens* linear electron flow components (white boxes) and key photoprotective mechanisms studied in this study. NPQ players (PSBS, LHCSR and VDE) are highlighted in orange. CEF‐mediated pathway components (NDH complex, PGRL1‐PGR5 complex) are highlighted in blue. FLVA/FLVB of PCEF pathway are indicated in pink. (B) Plants were initially grown at control light (CL, 50 μmol·photons·m^−2^·s^−1^) for 4 days before exposure to high light (HL, 500 μmol·photons·m^−2^·s^−1^) or fluctuating light (FL, 25/800 μmol·photons·m^−2^·s^−1^ 5/1 min) for 6 days. A control group of samples was also maintained under CL for 10 days.

Protonemal tissue of Gransden wild‐type (WT) ecotype of *P. patens* was grown on minimum PpNO_3_ medium solidified with 0.8% Agar (Ashton et al. 1979). Plants were propagated under sterile conditions on 9‐cm Petri dishes overlaid with a cellophane disk. Plates were placed in a growth chamber under controlled conditions: 22°C, 16‐h light/8‐h dark photoperiod, and a light intensity of 50 μmol·photons·m^−2^·s^−1^ (control conditions; CL). For excess light and fluctuating light acclimation, 4‐day‐old plants were moved for 6 days from control to 500 μmol·photons·m^−2^·s^−1^ (high light; HL) and 25/800 μmol·photons·m^−2^·s^−1^ for 5/1 min, respectively, (fluctuating light; FL), maintaining temperature and photoperiod. Photoprotective mutants (Figure 1A) used in this study were previously isolated. *psbs lhcsr1 lhcsr2* KO (hereafter referred to as *psbs lhcsr* KO for clarity) (Alboresi et al. 2010), *vde* KO (Pinnola et al. 2013), *pgrl1* KO (Kukuczka et al. 2014), *pgrl1 ndhm* KO (Storti et al. 2020b), *flva/b* KO (Gerotto et al. 2016; Traverso et al. 2025).

### 2.2 In Vivo Chlorophyll a Fluorescence and P700^+^ Measurement With Dual‐PAM

In Vivo chlorophyll *a* fluorescence and oxidised P700^+^ absorption signal were monitored simultaneously at room temperature with a Dual PAM‐100 fluorometer (Walz). Before measurements, plates were dark acclimated for 40 min. PSII and PSI parameters were calculated as following: Fv/Fm as (Fm − Fo)/Fm, NPQ as (Fm − Fm′)/Fm′, Y(II) as (Fm′ − F)/Fm′, Y(NO) as F/Fm, Y(NPQ) as F/Fm′‐F/Fm, qL = (Fm′‐F)/(Fm′‐Fo′) × Fo′/F, Y(I) as 1 − Y(ND) − Y(NA), Y(NA) as (Pm − Pm′)/Pm, Y(ND) as (1 − P700 red). Actinic light intensity (850 µmol·photons·m^−2^·s^−1^) was sub‐saturating for photosynthesis in WT plants grown under constant light (CL) (Gerotto et al. 2011).

### 2.3 Spectroscopic Analyses With Joliot‐Type Spectrometer (JTS)

Spectroscopic analysis was performed In Vivo on 10‐day‐old intact tissues using a JTS‐10 spectrophotometer (Biologic). Relative amount of functional photosynthetic complexes was evaluated by measuring the electrochromic shift (ECS) spectral change on buffer‐infiltrated plants (HEPES 20 mM, pH 7.5, KCl 10 mM) in the presence and absence of 3‐(3,4‐dichlorophenyl)‐1,1‐dimethylurea (DCMU 20 μM) and hydroxylamine (HA, 4 mM) as in previous studies (Bailleul et al. 2010; Gerotto et al. 2016). PSII functional antenna size was calculated by treating the sample with 3‐(3,4‐dichlorophenyl)‐1,1‐dimethylurea (DCMU 20 μM). The DCMU‐treated sample was exposed to actinic light 150 µmol·photons·m^−2^·s^−1,^ and PSII antenna size was estimated from *Fm* saturation kinetic (1/*t*_2/3_) (Cardol et al. 2008).

### 2.4 Total Protein Extracts

**Figure 2:**
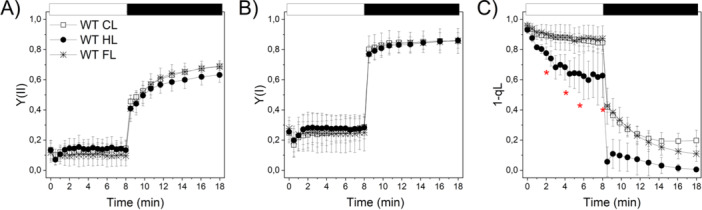
Photochemistry of acclimated WT *P. patens* at different light intensities. (A) PSII and (B) PSI quantum yield, and (C) PSII excitation pressure (1‐qL) were measured in plants acclimated to (CL), high light (HL) and fluctuating light (FL), represented by empty squares, black circles and stars, respectively. Plants were exposed to 850 µmol·photons·m^−2^·s^−1^ actinic illumination for 8 min, followed by 10 min of dark. Bars indicate standard deviation (*n* ≥ 3 biological replicates). Red asterisks indicate values significantly different from the CL sample (one‐way ANOVA, *p* < 0.01; statistical analysis considered time points at 0, 2, 4, 6 and 8 min after actinic light exposure). [Color figure can be viewed at wileyonlinelibrary.com]

Total extracts from *P. patens* grown in CL, HL and FL conditions were obtained by grinding tissues in sample buffer (50 mM TRIS pH 6.8, 100 mM DTT, 2% SDS, and 10% glycerol) before SDS/PAGE. Samples were loaded with the same equivalent amount of chlorophylls. For immunoblotting analysis, after SDS/PAGE, proteins were transferred to nitrocellulose membranes and detected with both Horseradish Peroxidase (HRP, Agrisera #AS09‐60s) or Alkaline Phosphatase‐conjugated secondary antibody (Sigma, #A3562) after hybridisation with specific primary homemade polyclonal antibodies (α‐LHCSR, α‐PSBS, α‐NDHM, α‐FLVB). For densitometry quantification of Western blot bands, images were processed with FIJI (https://fiji.sc/) using the ‘mean grey value’ for measurements. For each band, the relative background was also subtracted. Mean grey value was measured for each band and normalised to WT CL. Protein extracts from plants grown under the same light treatment were analysed at different dilutions using the same primary antibody. Each data point in the bar plot (Figure 2D) represents the average of replicates from a single independent experiment. For each antibody, at least three independent immunoblot experiments were performed. Chl *a/b* and Chl/Car ratios were obtained by fitting the spectrum of 80% acetone pigment extracts with spectra of the individual purified pigments, as reported in (Chazaux et al. 2022).

### 2.5 Growth Test

Growth on PPNO_3_ media under different light regimes (specific conditions are described in the text and in the figure legends) was evaluated starting from protonema colonies of 2 mm in diameter and then followed for 28 days. High‐resolution images (600 dpi) were acquired using a Konica Minolta Bizhub C280 scanner. Images were processed with FIJI (https://fiji.sc/) using the ‘threshold colour’ plugin to remove the plate background. Integrated density was measured for each colony and normalised to WT CL day 1 (Storti et al. 2019). At least three independent growth experiments were performed, one or two moss colonies per genotype and conditions were considered for each biological replicate.

### 2.6 Statistical Analysis

Descriptive statistics and inferential statistics were performed using OriginPro9.1 software. Differences between light treatments (CL, HL, FL) were statistically tested by one‐way ANOVA for WT characterisation. The effects of different mutations, light treatment, and their interaction were analysed using a two‐way ANOVA. A Tukey HSD post hoc test was performed to assess pairwise differences between genotypes. The number of biological replicates and significance levels are reported in each legend.

## 3 Results

### 3.1 Physcomitrium Patens Photosynthetic Apparatus Acclimates to Different Light Regimes

To investigate the mechanism of light acclimation in *P. patens*, WT plants were grown for 6 days under three distinct light conditions: CL, HL and FL (Figure 1B). Maximal PSII quantum efficiency (F_v_/F_m_) remained stable across the three conditions, indicating that the plants did not present extensive photodamage and thus were able to acclimate effectively to different light regimes (Table 1). Pigment extracts of acclimated plants revealed a lower Chl/car ratio under HL conditions as compared to CL and FL, suggesting an enhanced photoprotective role of carotenoids under HL. In contrast, the Chl *a/b* ratio remained unchanged across treatments. The PSII functional antenna size, estimated from fluorescence induction kinetics in the presence of DCMU (Cardol et al. 2008), was not significantly affected by the different light regimes in WT plants. This is consistent with earlier work showing limited modulation of antenna size in *P. patens* (Gerotto et al. 2011).

**Table 1:** Pigment composition and chlorophyll fluorescence analysis of acclimated WT plants, showing Chl/Car and Chl a/b ratios. Data represent mean values ± standard deviation.

	Chl/Car	Chl *a*/*b*	Antenna size	Fv/Fm
WT CL	3.7 ± 0.33	2.5 ± 0.15	0.025 ± 0.005	0.78 ± 0.01
WT HL	2.67^a^ ± 0.12	2.7 ± 0.34	0.026 ± 0.004	0.76 ± 0.03
WT FL	3.47 ± 0.16	2.7 ± 0.25	0.03 ± 0.005	0.77 ± 0.04

^a^ Statistically significant difference with respect to WT plants, one‐way ANOVA, *p* < 0.01, n > 5

To evaluate the response of key photosynthetic parameters after plant acclimation to different light regimes, we measured Chl *a* fluorescence and P700⁺ absorption signals under strong actinic illumination followed by dark relaxation. The overall efficiency of PSII (Y(II)) and PSI (Y(I)) was similar for plants grown in the three conditions (Figure 2A,B). Acclimation to HL increased the fraction of open PSII reaction centres upon actinic light, as shown by the decreased 1‐qL parameter (Figure 2C). This suggests that HL acclimated plants experience less PSII saturation (Kramer et al. 2004) once exposed to strong light, indicating enhanced photosynthetic electron transport capacity.

### 3.2 Acclimation to Variable Light Regimes Is Achieved Through Enhanced Photoprotective Mechanisms

**Figure 3:**
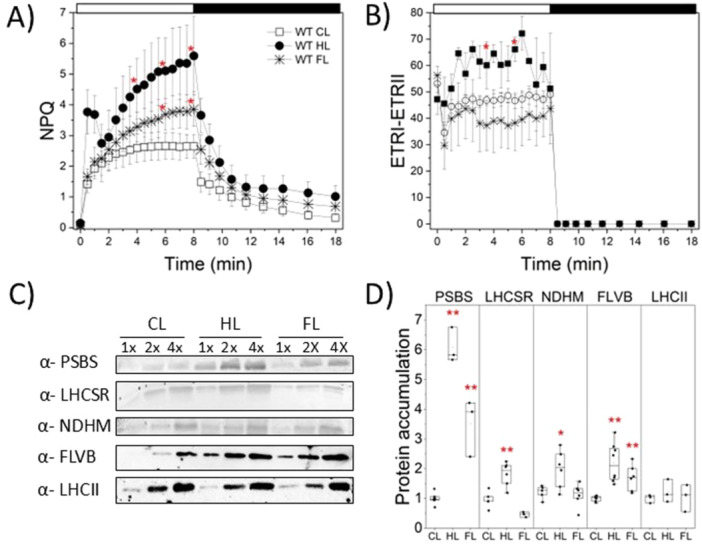
Functional and biochemical analysis of photoprotection related proteins in *P. patens* plants. (A) NPQ (B) ETRI‐ETRII. Plants were exposed for 8 min to actinic light (850 µmol·photons·m^−2^·s^−1^) followed by 10 min of darkness. Data are presented as empty squares (CL), black circles (HL), and stars (FL). Error bars indicate standard deviation (*n* ≥ 3 biological replicates). Red asterisks indicate values significantly different from the CL sample (one‐way ANOVA, *p* < 0.01. Minutes 0, 2, 4, 6, 8 after switching on the actinic light were considered for statistical analysis). (C) Immunoblot analysis of photoprotection‐related proteins in WT plants grown under CL, HL and FL. The proteins analysed included LHCSR, PSBS, FLVB, NDHM and LHCII. Protein loading corresponded to different chlorophyll equivalents as follows: 1× is equivalent to 1 µg of chlorophylls, and 2× and 4× indicate two‐ and fourfold amounts, respectively. (D) Quantification of PSBS, LHCSR, NDHM, FLVB, LHCII protein levels was densitometric analysis of immunoblots. Band intensities were normalised to CL‐grown plants present on the same membrane. Bar plots indicate mean (empty square) and median (horizontal lines within bars), with black circles represent individual biological replicate measurements (*n* ≥ 3). Red asterisks indicate statistically significant differences compared to CL condition (one‐way ANOVA, *p < 0.05, **p ≤ 0.01). [Color figure can be viewed at wileyonlinelibrary.com]

To investigate the regulation of photoprotection during long‐term acclimation, we assessed WT capacity to modulate NPQ and AET under different light conditions along with the biochemical accumulation of proteins involved in these mechanisms. NPQ activity varied depending on growth conditions, with HL‐acclimated plants showing NPQ levels twice as high as those grown under CL conditions. Similarly, plants exposed to FL showed an increased NPQ compared to CL (Figure 3A). The different electron transport rates between PSII and PSI were used to evaluate CEF around PSI. HL‐acclimated plants displayed higher CEF than those grown under CL and FL, as estimated from the difference between ETRI and ETRII (Figure 3B).

Immunoblot analysis showed that both PSBS and LHCSR, the molecular activators of NPQ in *P. patens* (Alboresi et al. 2010), were more abundant in HL compared to CL. PSBS was also more abundant in FL compared to CL (Figure 3C,D), in agreement with the enhanced NPQ during acclimation to HL and FL (Figure 3A). To investigate the regulation of alternative electron transporters, we examined the accumulation of the NDHM subunit (Storti et al. 2020a), an essential component of the NADH dehydrogenase‐like complex I. PGRL1 and PGR5 were not quantified because *Arabidopsis thaliana* antibodies did not recognise well *P. patens* proteins, while FLVB was chosen as a representative of PCEF (Gerotto et al. 2016). Both NDHM and FLVB accumulated more in HL‐acclimated plants compared to CL plants. Interestingly, FL acclimated plants showed a higher amount of FLVB than CL grown plants, but similar NDHM accumulation (Figure 3C,D). While the accumulation of proteins involved in photoprotection changed during acclimation, the same amount of Light Harvesting Complexes II (LHCII) was detected across the three conditions, consistent with pigment assessment and antenna size estimations.

### 3.3 NPQ and AET Mechanisms Are Critical for Optimal Growth During Acclimation to Different Light Regimes

Functional and biochemical analyses of WT *P. patens* plants revealed enhanced photoprotective mechanisms under challenging light environments. To better understand the roles of these mechanisms in acclimation, a set of photoprotective mutants was grown under the same CL, HL and FL conditions alongside WT plants. The mutants included those defective in NPQ and xanthophyll cycle (i.e., *vde* KO, *psbs lhcsr* KO) and in AET pathways (i.e., *pgrl1* KO, *pgrl1 ndhm* KO, *flva/b* KO).

**Figure 4:**
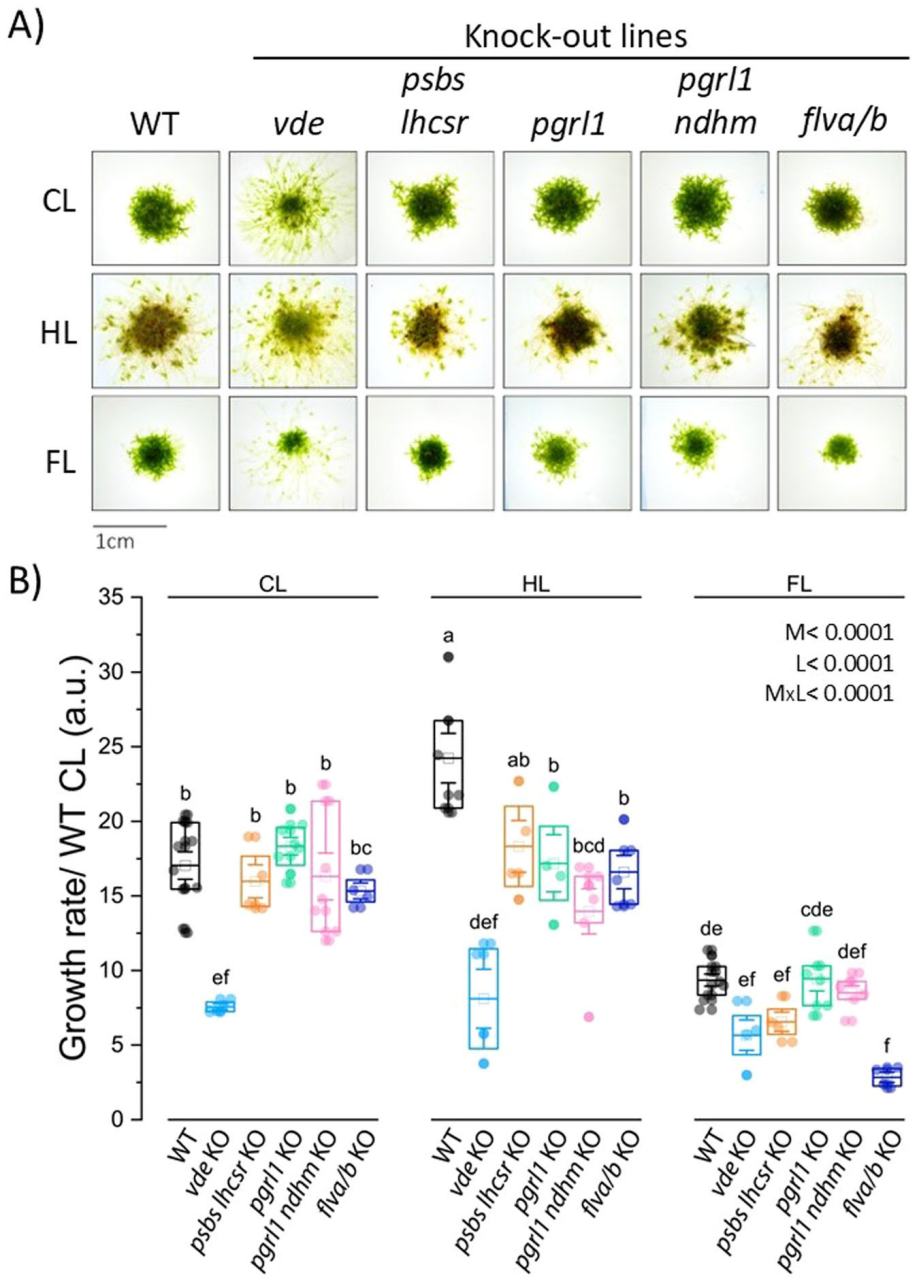
Growth phenotypes of *P. patens* plants after 28 days of growth under different light conditions. (A) Representative images of plants grown under CL, HL and FL conditions for 28 days. (B) Quantification of plant growth under each condition. Bar plots indicate mean (empty square) and median (horizontal lines within bars), circles represent individual samples (biological replicates *n* ≥ 3, see Section 2 for detailed information). For each biological replicate one or two individuals were considered for the analysis. WT, *vde* KO, *psbs lhcsr* KO, *pgrl1*, *pgrl1 ndhm, flva/b* KO are indicated in black, light blue, orange, green, pink and blue, respectively. Effects of light treatment (L), mutation (M) and their interaction (LxM) were tested by two‐way ANOVA. Results are reported at the top left of the figure. Values marked with different letters are significantly different between treatments (Tukey HSD post hoc test, *p* < 0.05). [Color figure can be viewed at wileyonlinelibrary.com]

Plant growth was monitored over a 28‐day period to assess growth differences, allowing the plant colonies to reach a diameter of ~1 cm. After 28 days, WT plants exhibited the highest growth under HL, while under FL, their growth decreased by 30% compared to CL (Figure 4). Under CL, all tested mutants, except *vde* KO, exhibited growth rates comparable to WT plants. In all tested conditions, the *vde* KO showed more dispersed protonemata development compared to WT, and a lack of response to HL and FL, highlighting the crucial role of zeaxanthin for plant growth and light acclimation. Under HL, all mutants, except *psbs lhcsr* KO, grew less than WT plants, with the mutant *pgrl1 ndhm* KO showing the most pronounced decrease (Figure 4). In FL conditions, *psbs lhcsr* KO, *pgrl1* KO, *pgrl1 ndhm* KO and *flva/b* KO lines displayed a significant growth reduction compared to CL. Moreover, in FL conditions *flva/b* KO showed a clear and significant growth impairment as compared to WT plants. Overall, all mutants showed some growth penalty, confirming that both NPQ and AET mechanisms are critical for optimal growth under different light regimes, with specific roles depending on the nature of the light stress encountered.

### 3.4 NPQ and AET Mechanisms Provide Critical Photoprotection in Variable Light Environments

To evaluate the impact of compromised photoprotection on photosynthetic efficiency, we measured key parameters in 10‐day‐old protonema of WT and photoprotective mutants grown under CL, HL and FL. The F_v_/F_m_ ratio, reflecting the maximum efficiency of PSII, remained stable across the three light regimes in WT plants, revealing its photosynthetic acclimation capacity.

**Figure 5:**
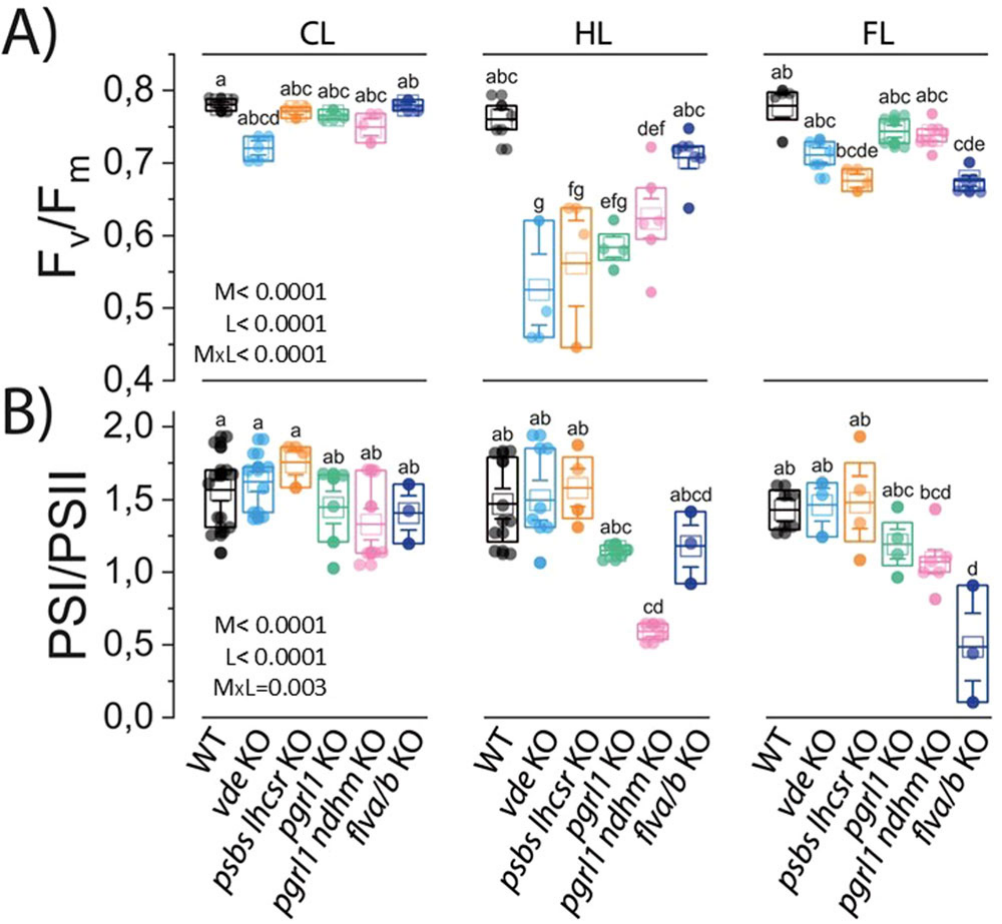
PSII maximal efficiency and PSI/PSII ratio in *P. patens* plants. (A) PSII maximal quantum efficiency (F_v_/F_m_) of WT and mutant lines (*vde* KO, *psbs lhcsr* KO, *pgrl1* KO, *pgrl1 ndhm* KO, *flva/b* KO) grown under CL, HL and FL. (B) PSI/PSII ratio quantified from ECS signals following the application of a flash of light on the samples. For each condition, mean and median are shown, respectively, with an empty square and horizontal lines in the bar blot. Different biological replicates are indicated with points (*n* ≥ 3). WT, *vde* KO, *psbs lhcsr* KO, *pgrl1*, *pgrl1 ndhm, flva/b* KO are indicated in black, light blue, orange, green, pink and blue, respectively. Genotypes conditions are reported at the bottom of panel (B). Effects of light treatment (L), mutation (M) and their interaction (LxM) were tested by two‐way ANOVA. Results are shown in the bottom right corner of each panel. Values marked with different letters are significantly different between treatments (Tukey HSD post hoc test, *p* < 0.05). [Color figure can be viewed at wileyonlinelibrary.com]

Under CL conditions, all mutant genotypes were similar to WT (Figure 5A). Interestingly, acclimation to HL and FL reduced F_v_/F_m_ across all mutants relative to WT plants grown under the same conditions, suggesting the presence of light‐induced damage. Notably, mutants deficient in NPQ (*psbs lhcsr* KO, *vde* KO) and in CEF (*pgrl1* KO, *pgrl1 ndhm* KO) displayed the lowest PSII efficiency under HL, whereas PCEF mutant *flva/b* KO exhibited the most significant reduction under FL (Figure 5A).

We also evaluated the relative activity of the two PS using ECS signal analysis following a single flash of light (Bailleul et al. 2010) (Figure 5B). Under CL conditions, all lines showed PSI/PSII ratios like WT. However, under HL and FL conditions, while NPQ mutants retained a PSI/PSII ratio comparable to WT plants, the AET mutants showed reduced ratios, likely as a result of PSI inactivation. In particular, the double mutant *pgrl1 ndhm* KO showed a marked reduction in PSI/PSII ratio under HL. Under FL conditions, the PSI/PSII ratio also decreased in the *pgrl1 ndhm* KO mutant, though the reduction was less severe than under HL. Conversely, pseudo‐cyclic *flva/b* KO mutant displayed the most significant decline in the PSI/PSII ratio under FL conditions.

### 3.5 Efficient Energy Partitioning Between Photosystems Requires Effective Photoprotection During Light Acclimation

**Figure 6:**
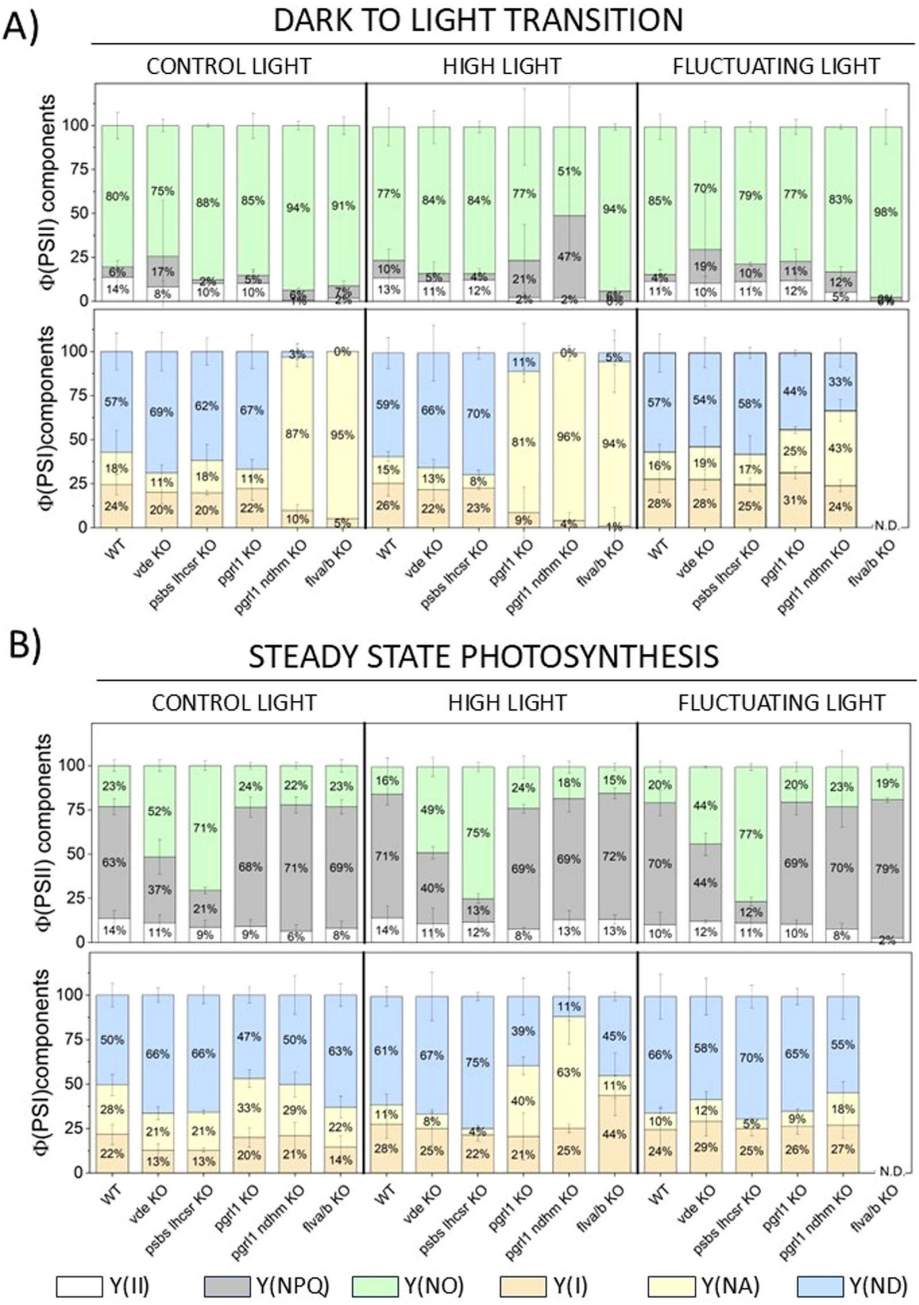
PSII and PSI energy partitioning in WT and photo‐protective mutants acclimated to different light conditions. (A) Parameters measured during the light‐to‐dark transition at the onset of actinic illumination, and (B) parameters measured during steady‐state photosynthesis (minutes 5–8 of actinic illumination) in WT and mutant plants acclimated to control light (CL), high light (HL), and fluctuating light (FL). Acclimated plants were exposed to 850 µmol·photons·m^−2^·s^−1^ actinic light illumination using a DUAL‐PAM chlorophyll fluorometer. Data represent the mean of at least 3 biological replicates ± SD (see Supporting Information S1: Figures for the full dataset). Parameters include Y(II), PSII quantum yield (white); Y(NPQ), non‐photochemical quenching (grey); Y(NO), non‐regulated energy dissipation (green); Y(I), PSI quantum yield (orange); Y(NA), PSI acceptor‐side limitation (yellow); Y(ND), PSI donor‐side limitation (blue). N.D. = not detectable. Each individual graph in the Supporting Information S1: Figures includes the corresponding statistical tests.

Given that impaired photoprotection can affect photosystem efficiency and activity, we investigated the contributions of NPQ and AET to energy allocation within PSII and PSI. Using a DUAL‐PAM‐100, we simultaneously monitored Chl *a* fluorescence and P700^+^ absorption signals during 8‐min exposure to actinic light, followed by 10 min of dark relaxation as in Figure 2. Our analysis focused on two critical time points: (i) the dark‐to‐light transition, to assess the impact of NPQ and AET upon a sudden increase in illumination (Figure 6A), and (ii) steady‐state photosynthesis after 5–8 min of illumination, when the Calvin‐Benson Cycle is fully activated (Figure 6B). At the onset of light, in the WT around 14% of energy was involved in photochemistry (Y(II)), 6% was dissipated as heat (Y(NPQ) and 80% of the energy was dissipated at the level of reaction centres in non‐regulated manner (Y(NO)), a component that represents the constitutive thermal dissipation that occurs independently of protective regulatory mechanisms. Moreover, 57% of PSI were donor‐side limited (Y(ND)) and 18% were acceptor‐side limited (Y(NA)), resulting in 24% of PSI that could be photo‐oxidised with a saturating pulse (YI). When the light was switched on, NPQ mutants showed similar energy allocation between the photosystems compared to WT, likely because NPQ requires several minutes to become fully activated (Figure 6A, Supporting Information S1: Figure S1–S2). In contrast, AET mutants tended to display differences during the dark‐to‐light transition. Specifically, *pgrl1 ndhm* KO and *flva/b* KO mutants demonstrated reduced Y(II) efficiency in all growth conditions (0%–5%) compared to approximately 12% in WT, with *pgrl1* KO mutants under HL showing similar reductions (around 2%). Furthermore, these AET mutants enhanced energy dissipation via either Y(NO) or Y(NPQ), depending on the growth conditions (Figure 6A, Supporting Information S1: Figure S3). Impaired photoprotection also impacted PSI energy allocation during the light‐to‐dark transition in AET mutants (Supporting Information S1: Figure S4). *pgrl1 ndhm* KO lines displayed higher Y(NA) values under both CL (87%) and HL (96%) conditions compared to WT plants (CL: 18%, HL: 15%). Although this difference persisted under FL, it was less pronounced (WT: 16%, *pgrl1 ndhm* KO: 43%). Notably, the single *pgrl1* KO mutant showed a milder phenotype, with high Y(NA) values observed only under HL (81%). These results indicate that under high light conditions, both the NDH and the PGRL1/PGR5 pathway are required to maintain proper PSI functionality upon light exposure. In the *flva/b* KO mutant, Y(NA) reached maximal levels at the onset of light under CL (95%) and HL (94%). In plants grown under FL, the absence of FLVs resulted in such severe PSI impairment that accurate energy allocation measurements were not possible, and overall yield was drastically reduced (Figure 6A).

During steady state photosynthesis, when the Calvin‐Benson Cycle is fully active, WT plants displayed maximal NPQ activation (Y(NPQ), ~63%) and minimal non‐regulated energy dissipation (Y(NO), ~23%), while similar energy allocation within PSI was observed compared to the dark to light transition, consistent with the very fast activation of AET.

In these conditions, both NPQ and AET mutants displayed trends in energy partitioning between photosystems compared to WT (Figure 6B). Under CL conditions, the Y(NPQ) was reduced in *vde* KO plants (37%) compared to WT (63%), and even further decreased in *psbs lhcsr* KO plants (21%) (Figure 6B, Supporting Information S1: Figure S1). This reduction was compensated by a concomitant increase in the constitutive loss process Y(NO) in these mutants. Notably, this energy distribution pattern was maintained in WT and *vde* KO lines acclimated to HL and FL. In contrast, *psbs lhcsr* KO mutants experienced a further decrease in Y(NPQ) accompanied by a increase in Y(NO) under HL and FL conditions (Figure 6B, Supporting Information S1: Figure S1). Regarding PSII energy partitioning, the *pgrl1* and *pgrl1 ndhm* mutants displayed profiles similar to WT under all conditions. However, under FL, the absence of FLVs in the *flva/b* KO mutant significantly reduced the Y(II) efficiency (2%) and caused alterations in the development and relaxation of Y(NPQ) (Figure 6B, Supporting Information S1: Figure S3).

At PSI, impaired photoprotection also led to altered energy allocation during steady‐state photosynthesis. NPQ mutants showed increased donor side limitation during actinic light exposure, with maximal increase observed in HL acclimated plants (Figure 6B, Supporting Information S1: Figure S2). This suggests that in these mutants, inefficient thermal energy dissipation impaired linear electron transport, reducing the number of electrons reaching PSI and thereby limiting the availability of electrons to PSI. Moreover, under HL conditions, at steady state photosynthesis, the *pgrl1* and *pgrl1 ndhm* KO lines exhibited high values of acceptor side limitation (40% in *pgrl1* KO and 63% in *pgrl1 ndhm* KO, compared to 11% in WT) and minimal donor side limitation (39% in *pgrl1* KO and 11% in *pgrl1 ndhm* KO, compared to 61% in WT) (Figure 6B, Supporting Information S1: Figure S4). Under other growth conditions, these CEF mutants displayed values similar to WT. In contrast, the *flva/b* KO mutants appear to have similar PSI energy partitioning under steady‐state conditions across all light treatments tested (CL and HL), suggesting that when the Calvin‐Benson cycle is fully functional, alternative mechanisms help safeguard PSI.

## 4 Discussion

### 4.1 Enhancing Photoprotection to Maintain Photosynthetic Efficiency

This study explored the strategies that *P. patens* plants employ to sustain photosynthetic efficiency during long‐term acclimation to HL and FL. The use of *P. patens* as a model organism allowed us to study the effects of multiple photoprotective mechanisms simultaneously, some of which are shared with both green algae and vascular plants. The study of bryophytes also provides insights on the adaptation of the early plants that transitioned from aquatic to terrestrial environments and were confronted with harsh conditions, including water loss and high levels of sunlight (Proctor, MCF 2014). Survival in such an environment would have been impossible without effective strategies to protect against light‐induced stress. In this context, our findings highlight that the activation of distinct mechanisms enables *P. patens* to cope with adverse light regimes, thereby optimising photosynthetic efficiency and energy distribution along the electron transport chain.

We showed that upon exposure to HL and FL, WT *P. patens* retained F_v_/F_m_ and efficient functioning of both photosystems, demonstrating that it is able to effectively acclimate to those challenging conditions (Table 1, Figure 2). The conditions chosen were effectively stressful for the moss, as shown by the growth defects of mutant lines (Figure 5) indicating that acclimation is indeed effective in WT plants.

Unlike other photosynthetic organisms (Walters and Horton 1994; Ballottari et al. 2007; Jia et al. 2016; Meneghesso et al. 2016; Štroch et al. 2022), *P. patens* does not primarily rely on antenna size adjustment for light acclimation. This observation aligns with previous studies in *P. patens* under HL and low temperature (Gerotto et al. 2011), in *C. reinhardtii* under HL (Bonente et al. 2012) and in *Picea abies* (Štroch et al. 2022). HL‐acclimated plants exhibited increased carotenoid accumulation, a conserved strategy to prevent photo‐oxidative damage (Havaux 2014). Conversely, no significant changes in pigment composition were observed between FL‐ and CL‐grown plants, consistent with *A. thaliana* responses to fluctuating light (Gollan et al. 2023) (Table 1).

Long‐term acclimation to both HL and FL induced an increased accumulation of proteins involved in photoprotection (Figure 3). Both light regimes triggered NPQ upregulation, with HL‐acclimated plants exhibiting the highest NPQ levels. Upregulation of NPQ during excess light exposure is well‐conserved in the green lineage and has been extensively observed and studied in many organisms, including green algae such as *Chlamydomonas reinhardtii* and vascular plants like *Arabidopsis thaliana* (Peers et al. 2009; Ware et al. 2015; Flannery et al. 2021). In *P. patens* NPQ upregulation under HL is associated with enhanced accumulation of LHCSR and even higher accumulation of PSBS (Gerotto et al. 2011). Our data indicates that FL triggers a similar response, characterised by elevated NPQ and increased PSBS levels, suggesting that rapid NPQ activation protects plants from sudden light excess‐ a response that appears to be conserved in angiosperms. For instance, *A. thaliana* plants deficient in NPQ photoprotection exhibit growth defects under fluctuating light conditions in a controlled environment (Külheim et al. 2002). NPQ also protects leaves of *N. tobacco* plants when exposed to natural sunlight and thus shaded by clouds or other leaves (Kromdijk et al. 2016). Furthermore, while under control light conditions, NPQ in *P. patens* depends more on LHCSR than PSBS (Alboresi et al. 2010), the acclimation to HL and FL shifts this reliance toward PSBS. This suggests that PSBS is more finely regulated in response to dynamic light environments. These findings support a model in which terrestrial plants, frequently exposed to variable light due to cloud cover and canopy movements, have undergone a functional shift from LHCSR‐ to PSBS‐dependent NPQ. The presence of both components in *P. patens* reveals an intermediate evolutionary stage, illustrating the gradual replacement of LHCSR by PSBS.

Our findings also indicated enhanced PSI photoprotection during acclimation to both HL and FL by modulation of AET mechanisms. However, while the levels of FLVB protein are increased in both conditions, the NDHM subunit representative of NDH is more accumulated only under HL, suggesting a major role of the CEF‐mediated pathway under this light regime. This finding aligns with earlier analyses of *P. patens* mutants (Storti et al. 2019). Overall, our results indicate that acclimation to HL and FL enhances photoprotection, allowing the maintenance of photosynthetic efficiency under different light conditions. Under all tested conditions, photoprotection of PSII depended on enhanced NPQ, while PSI photoprotection revealed an interplay between cyclic and pseudo‐cyclic electron flow, with the former appearing to have a major role under HL and the latter under FL conditions. However, as suggested by the analysis of CEF and PCEF mutants, CEF cannot fully compensate for the absence of FLVs under HL, nor can FLV cover for the lack of CEF under FL.

### 4.2 Effective Photosynthesis and Growth Depend on the Interplay of Multiple Photoprotective Mechanisms

The use of photoprotective mutants in *P. patens* enabled the evaluation of the contributions of NPQ and AET to growth and photosynthesis under different light conditions. The *vde* KO mutant showed severe growth defects even under CL (Figure 4) (Pinnola et al. 2013), likely due to the absence of zeaxanthin rather than solely due to impaired NPQ. Indeed, the *psbs lhcsr* KO mutant, which completely lacks NPQ, displayed a less severe growth phenotype compared to the *vde* KO under all analysed conditions. This observation contrasts with findings in both the green alga *C. reinhardtii* and the vascular plant *A. thaliana*, where growth defects have not been reported in *vde* KO mutants under various light conditions (Niyogi et al. 1997; Li et al. 2009a). Future experiments could investigate a specific dependence on zeaxanthin in *P. patens*, potentially linked to an altered xanthophyll cycle that affects carotenoid biosynthesis and hormone metabolism (Fujita et al. 2013; D'Alessandro and Havaux 2019), leading to an alteration of moss growth.

The absence of an efficient NPQ mechanism led to photoinhibition during acclimation, as indicated by a marked reduction in the F_v_/F_m_ parameter in *npq* mutants (Figure 5), a feature conserved in both *C. reinhardtii lhcsr* KO (Allorent et al. 2013) and *A. thaliana psbs* KO plants when grown under HL (Yang et al. 2022). A difference in photoinhibition can also be inferred by the changes in energy distribution at the level of PSII (Supporting Information S1: Figure S1). However, the absence of functional NPQ appears to be partially compensated by higher PSII repair rate (Roach and Krieger‐Liszkay 2012; Barbato et al. 2020), which helps maintain PSI/PSII values in NPQ mutants similar to WT plants (Figure 5). Both the NPQ mutants exhibited elevated donor side limitation (Y(ND)) during steady state during acclimation to HL and FL (Figure 6). This increase is likely resulting from mutants' inability to effectively manage excitation energy at the level of PSII, which can alter LEF and enhance electron transfer to PSI.

We also observed multiple interactions between AET pathways and the regulation of photosynthesis during acclimation to different light conditions, as evidenced by the phenotypes of *pgrl1* KO, *pgrl1 ndhm* double KO, and *flva/b* KO mutants. It has previously been shown that the relative contribution of the PGR5–PGRL1–mediated pathway to CEF is larger than that of the NDH‐dependent pathway in both mosses and angiosperms (Munekage et al. 2004; Storti et al. 2020a). Furthermore, the NDH‐1 complex is absent in some green algae, such as *C. reinhardtii*, and in certain gymnosperms (Ruhlman et al. 2015; de Vries et al. 2016). Based on this, we selected the *pgrl1* single KO and the *pgrl1 ndhm* double KO mutants as representative models of impaired CEF. Consistent with the biochemical analysis on WT plants, we observed that the lack of CEF caused the most pronounced acceptor side limitation in plants acclimated to HL. CEF mutants exhibited the highest levels of photoinhibition and the largest imbalance between the two photosystems under HL conditions. However, even under FL, they did not fully recover PSII maximal quantum efficiency and the number of functional photosystems, as indicated by F_v_/F_m_ and PSI/PSII parameters, respectively. This suggests that PCEF alone cannot fully offset the absence of CEF under these conditions (Figure 5).

The lowest photosynthetic activity observed in CEF mutants under HL was reflected in their growth performance, as they showed the most pronounced growth defects compared to the WT under the same conditions (Figure 4). CEF transport under HL appears to be particularly critical in *C. reinhardtii*; indeed, the *pgrl1* mutant was unable to acclimate photo‐autotrophically under 500 µmol·photons·m^−2^·s^−1^ and its growth was severely reduced compared to WT (Yadav et al. 2020). Under mild FL conditions (20/200), *C. reinhardtii pgrl1* KO displayed a growth rate comparable to WT, while under more severe FL (20/600), it displayed a clear growth reduction relative to WT (Jokel et al. 2018). In contrast, the *pgrl1ab* mutation in *A. thaliana* is lethal under FL (Rühle et al. 2021), highlighting a stronger dependency on CEF in vascular plants in this condition. This suggests that PGR5‐PGRL1‐mediated CEF acquired specific functions in algae, mosses, and vascular plants to respond to fluctuations in light intensity, partially compensating for the absence of FLV function. Once again, *P. patens* exemplifies the coexistence of distinct photoprotective strategies. The functional divergence observed between *C. reinhardtii* and *A. thaliana* points to its intermediate evolutionary position.

Under FL conditions, FLV activity is critical for growth in *P. patens*. *flv* KO mutants exhibited dramatically reduced growth under FL, while the phenotype was milder under HL, where the CEF can partially compensate for the absence of FLV (Figure 4) (Gerotto et al. 2016). A similar pattern was also observed in *C. reinhardtii flv* KO mutants, indicating a conserved role of FLVs in coping with light fluctuations (Chaux et al. 2017).

Furthermore, *P. patens* damage to PSI due to the absence of PCEF under FL was so severe that it was not possible to detect its activity (Figure 6). Additionally, the lack of PCEF also led to altered NPQ dynamics, impairing both its activation and relaxation, suggesting that proper electron flow downstream PSI is also essential for optimal energy management and dissipation at the level of PSII (Supporting Information S1: Figure S4).

Overall, our findings suggest that the efficient photosynthesis under varying light conditions is not dependent on a single mechanism but rather on the additive effect of multiple protective strategies. During long‐term acclimation, removing any one of these strategies leads to reduced energy efficiency and compromised growth. Indeed, in mutants defective in either NPQ (*vde* KO, *psbs lhcsr* KO) or AET (*pgrl1* KO, *pgrl1 ndhm* KO, *flva/b* KO) growth (Figure 4) and photosynthesis were impaired (Figure 5) under challenging light conditions. The biochemical and functional characterisation of WT and mutants during acclimation underscored that different photoprotective mechanisms operate in an additive manner to ensure proper energy management, with no single mechanism fully able to compensate for the absence of the other.

## Conflicts of Interest

The authors declare no conflicts of interest.

## Supporting information

Beraldo et al Supplemental Information PCE R1.

## Data Availability

The data that support the findings of this study are available from the corresponding author upon reasonable request.
