# Supplementary material for: Additive Effects of Multiple Photoprotective Mechanisms Drive Efficient Photosynthesis Under Variable Light Conditions
Source: Plant Cell Environ. 2025 Jun 17;48(10):7186–98. doi: 10.1111/pce.70016 (PMC12415417; doi:10.1111/pce.70016)
Supplement: Supplementary file 1 — Beraldo et al Supplemental Information PCE R1. [file PCE-48-7186-s001.pdf]

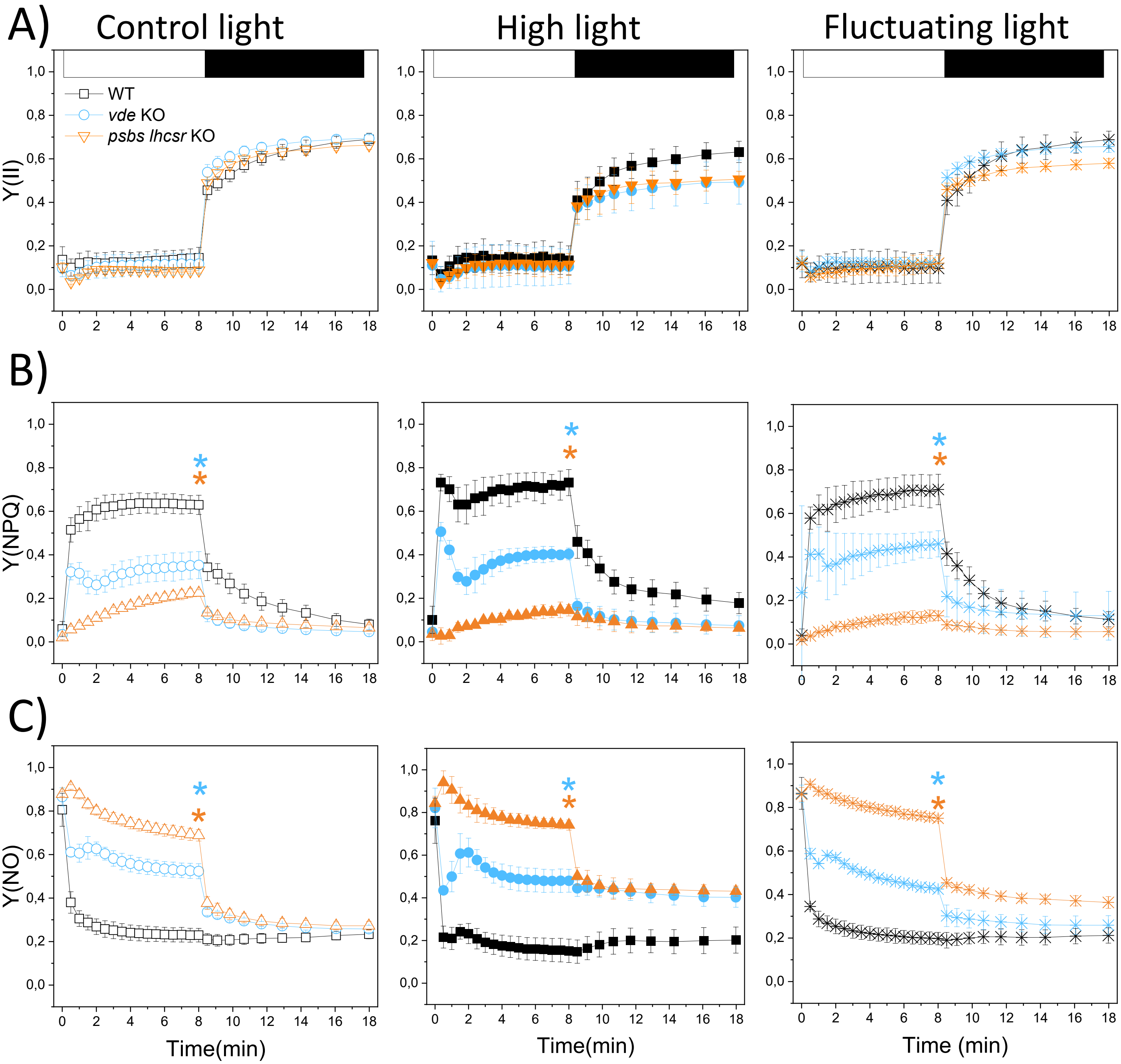

Figure S1. PSII efficiency of WT and NPQ mutants during light acclimation. A) Y(II), B) Y(NPQ), C) Y(NO). Eleven-days old protonema was treated 850 µmol of photons m^{-2} s^{-1} of actinic light for 8 minutes followed by 10 minutes of dark. Exposure to actinic irradiance is indicated at the top of the panels. Data for control light (CL), high light (HL) and fluctuating light (FL) are shown, respectively, in empty squares, black circles and stars. Bars indicate standard deviation ($n \geq 3$). WT, *vde* KO, *psbs lhcsr* KO are indicated in black, light blue and orange. Asterisks indicate significant differences between WT and mutants (One-Way ANOVA p<0,05). Timepoint 1 second and 8 minutes were considered for statistical analysis.

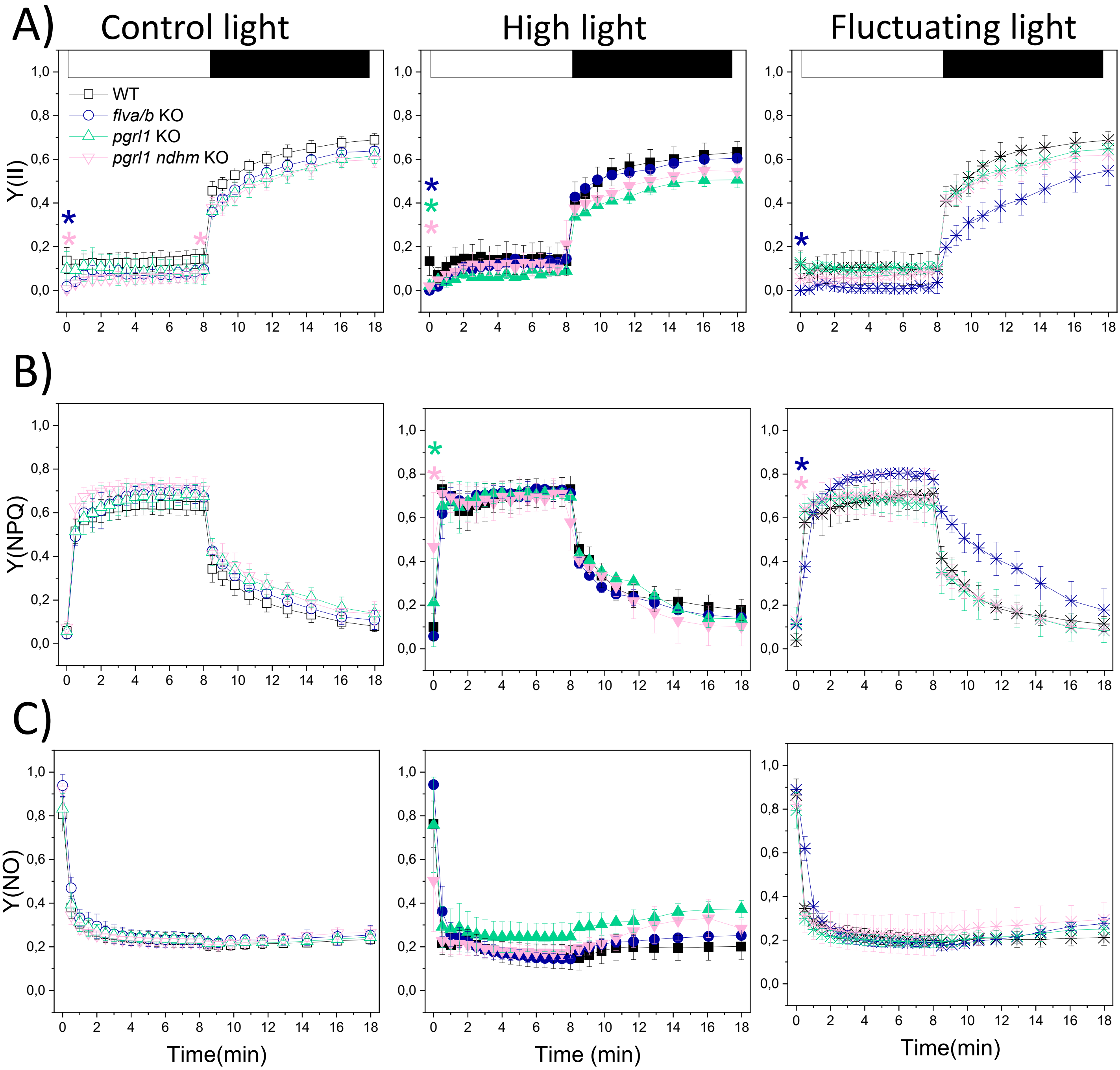

Figure S2. PSII efficiency of WT and photoprotective mutants during light acclimation. A) NPQ mutants B) AET mutants. Eleven-days old protonema was treated 850 µmol of photons m^{-2} s^{-1} of actinic light for 8 minutes followed by 10 minutes of dark. Exposure to actinic irradiance is indicated at the top of the panels. Data for control light (CL), excess light (HL) and fluctuating light (FL) are shown, respectively, in empty squares, black circles and half-up triangles. Bars indicate standard deviation ($n \geq 3$). WT, *vde* KO, *psbs lhcsr* KO, *pgrl1*, *pgrl1 ndhm, flva/b* KO are indicated in black, light blue, orange, green, pink and blue respectively. Asterisks indicate significant differences between WT and mutants (One-Way ANOVA p<0,05). Timepoint 1 second and 8 minutes were considered for statistical analysis.

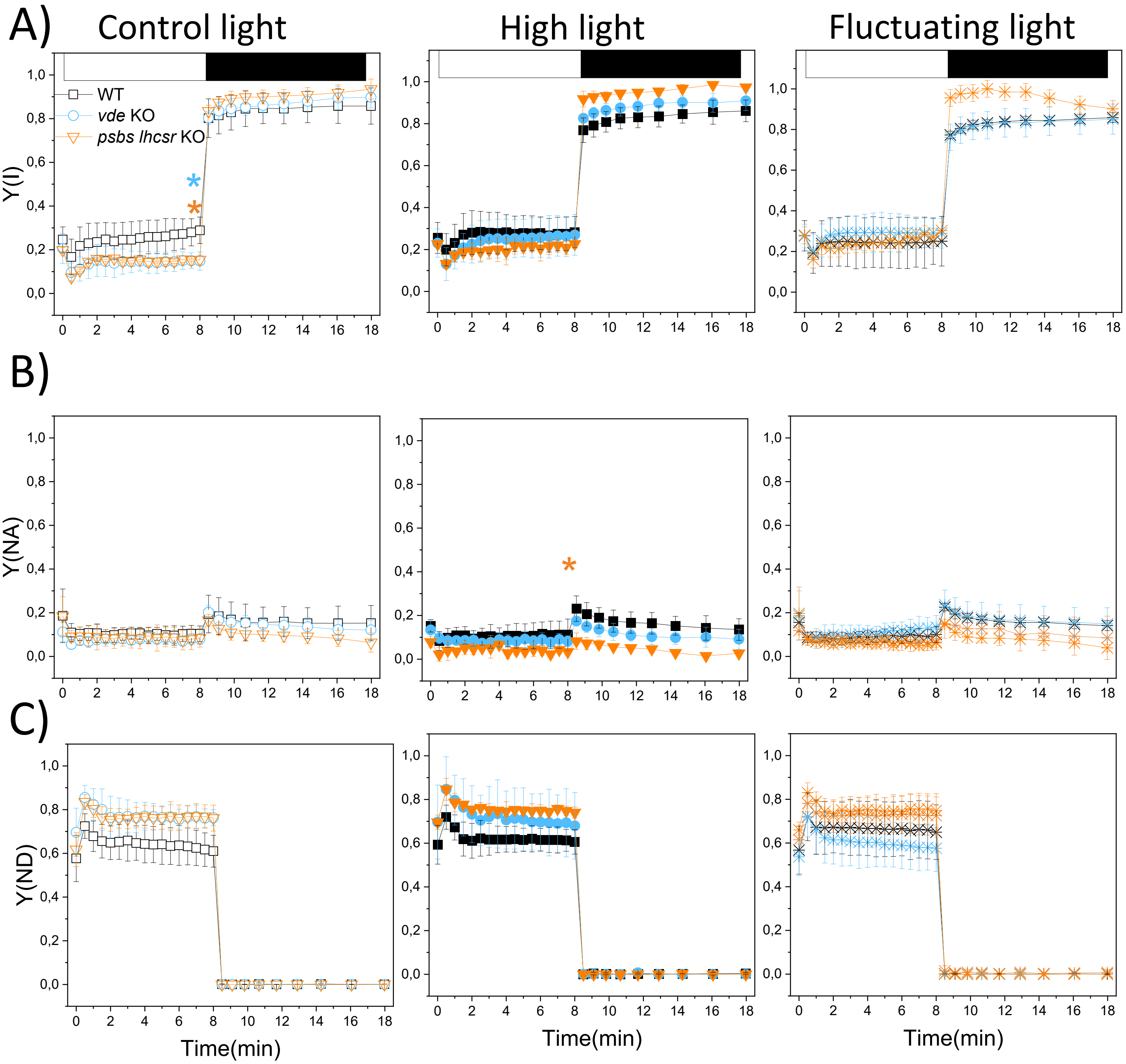

Figure S3. PSI properties of acclimated NPQ mutants to different light regimes. A) Y(I) B) Y(NA), C) Y(ND). Eleven-days old protonema was treated 850 µmol of photons m^{-2} s^{-1} of actinic light for 8 minutes followed by 10 minutes of dark. Exposure to actinic irradiance is indicated at the top of the panel. Data for control light (CL), high light (HL) and fluctuating light (FL) plants are shown, respectively, in empty squares, black circles and half-up triangles. Bars indicate standard deviation ($n \geq 3$). Wild-type, *vde* KO, *psbs lhcsr1 lhcsr 2* KO are indicated in black, light blue and orange, respectively. Asterisks indicate significant differences between WT and mutants (One-Way ANOVA p<0,05). Timepoint 1 second and 8 minutes were considered for statistical analysis.

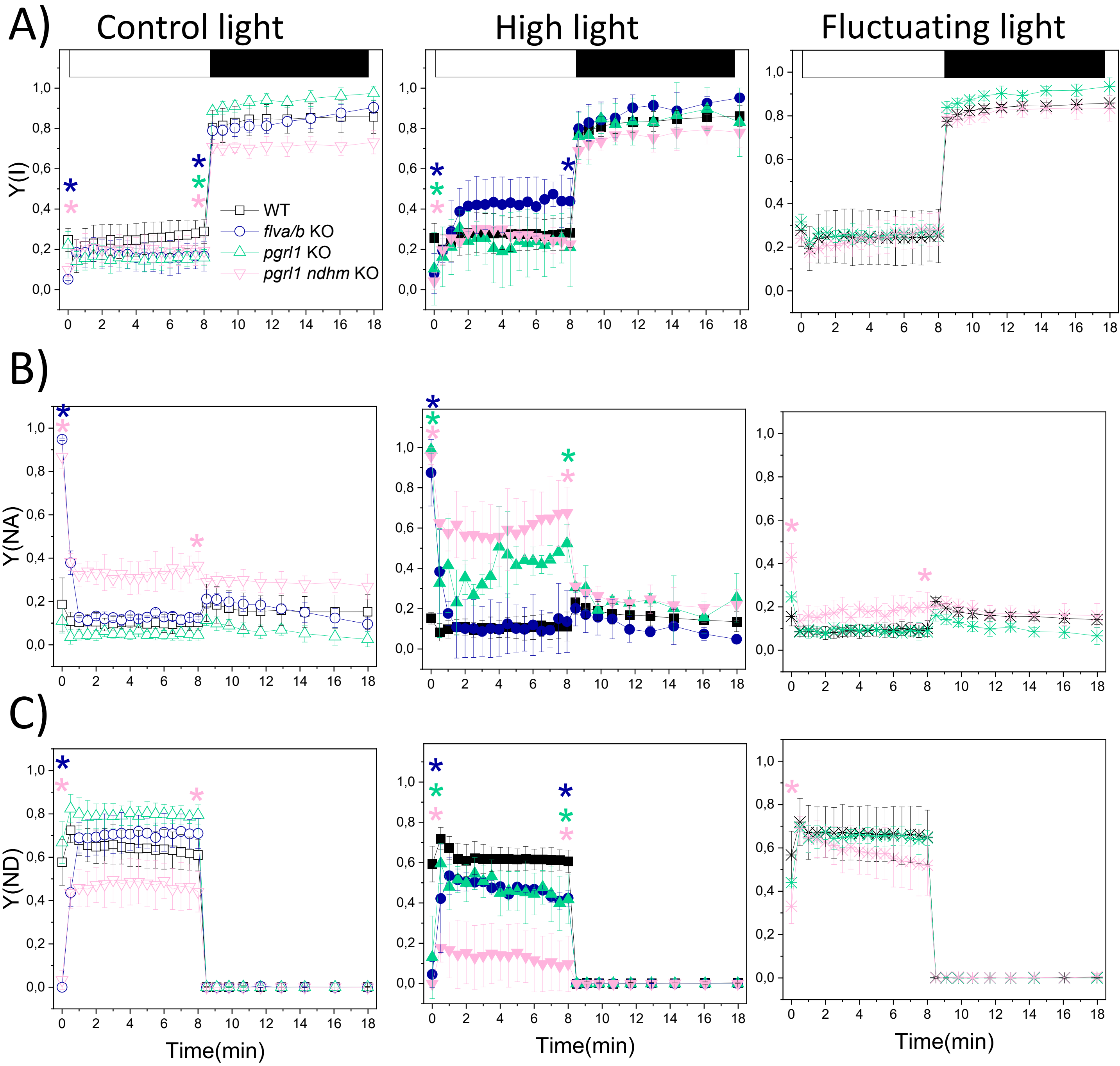

Figure S4. PSI properties of acclimated NPQ mutants to different light regimes. A) Y(I) B) Y(NA), C) Y(ND). Eleven-days old protonema was treated 850 µmol of photons m^{-2} s^{-1} of actinic light for 8 minutes followed by 10 minutes of dark. Exposure to actinic irradiance is indicated at the top of the panels. Data for control light (CL), high light (HL) and fluctuating light (FL) plants are shown, respectively, in empty squares, black circles and half-up triangles. Bars indicate standard deviation ($n \geq 3$). Wild-type, *flva/b* KO, *pgrl1* KO, *pgrl1 ndhm* KO are indicated in black, blue, green and pink respectively. *flva/b KO* under FL conditions was undetectable. Asterisks indicate significant differences between WT and mutants (One-Way ANOVA p<0,05). Timepoint 1 second and 8 minutes were considered for statistical analysis.
